# Gene expression links functional networks across cortex and striatum

**DOI:** 10.1038/s41467-018-03811-x

**Published:** 2018-04-12

**Authors:** Kevin M. Anderson, Fenna M. Krienen, Eun Young Choi, Jenna M. Reinen, B. T. Thomas Yeo, Avram J. Holmes

**Affiliations:** 1 Department of Psychology, Yale University, New Haven, CT 06520 USA; 2 Department of Genetics, Harvard Medical School, Boston, MA 02114 USA; 3 Department of Neurosurgery, Stanford University, Stanford, CA 94305 USA; 4 Department of Electrical and Computer Engineering, Clinical Imaging Research Centre, Singapore Institute for Neurotechnology and Memory Network Programme, National University of Singapore, Singapore, 117456 Singapore; 5 Athinoula A. Martinos Center for Biomedical Imaging, Massachusetts General Hospital, Boston, Charlestown, MA 02129 USA; 6 Department of Psychiatry, Yale University, New Haven, CT 06520 USA; 7 Department of Psychiatry, Massachusetts General Hospital, Boston, MA 02114 USA

## Abstract

The human brain is comprised of a complex web of functional networks that link anatomically distinct regions. However, the biological mechanisms supporting network organization remain elusive, particularly across cortical and subcortical territories with vastly divergent cellular and molecular properties. Here, using human and primate brain transcriptional atlases, we demonstrate that spatial patterns of gene expression show strong correspondence with limbic and somato/motor cortico-striatal functional networks. Network-associated expression is consistent across independent human datasets and evolutionarily conserved in non-human primates. Genes preferentially expressed within the limbic network (encompassing nucleus accumbens, orbital/ventromedial prefrontal cortex, and temporal pole) relate to risk for psychiatric illness, chloride channel complexes, and markers of somatostatin neurons. Somato/motor associated genes are enriched for oligodendrocytes and markers of parvalbumin neurons. These analyses indicate that parallel cortico-striatal processing channels possess dissociable genetic signatures that recapitulate distributed functional networks, and nominate molecular mechanisms supporting cortico-striatal circuitry in health and disease.

## Abstract (web summary)

The functional connectivity of brain regions can be reflected in a shared molecular architecture. This cross-modal study demonstrates correspondence of spatial patterns of gene expression to limbic and somato/motor cortico-striatal networks in human and non-human primates.

## Introduction

A fundamental challenge in neuroscience is to understand how brain activity is synchronized across large-scale networks. Coordinated patterns of spontaneous neural activity, measured through associated changes in blood flow with functional magnetic resonance imaging (fMRI), organize brain regions into functional networks supporting broad domains of cognition and behavior. Although network architecture is heritable^1,2^, predictive of behavior^3^, and disrupted in psychiatric illness^4^, the molecular mechanisms supporting functional organization remain poorly understood. The translational challenge of linking molecules to networks is addressable, in part, by integrating whole-brain transcriptional data with estimates of brain function^5^. To date, studies associating gene expression to functional networks have been largely constrained to cerebral cortex, likely to be due to the divergent cellular, molecular, and functional properties of cortex relative to the rest of the brain^5–8^. However, the organization of cortical networks is reflected in non-cortical structures such as the striatum^9^, suggesting the possibility of shared molecular-genetic properties distributed throughout the brain. A key question is whether patterns of gene expression show similarities across brain structures that are functionally connected but spatially and anatomically distinct, such as the cortex and striatum. Shared enrichment of genes among cortical and subcortical components of a network could reveal the network-preferential presence of a particular cell type, or enhanced sensitivity to a class of neurotransmitter, which would yield deep insight into underlying biological mechanism.

The human striatum receives widespread inputs from the cerebral cortex, forming connections that are central for motivated behavior, learning, and movement^10–13^. The influence of heritable factors on cortico-striatal circuits is evident from common genetic variants that contribute to subtle shifts in striatal reactivity^14^ and associated behaviors (e.g., reinforcement learning)^15^. Understanding the genetic mechanisms supporting cortico-striatal networks is pressing given their role in neurodevelopmental, psychiatric, and movement disorders marked by dysregulated incentive-based learning, goal-directed action, and habit formation^16–18^. Although decades of research has characterized the structural and functional architecture of cortico-striatal circuits^9–13,19–23^, relatively little is known about the molecular genetic associates of their network-level organization. Here we present the first comprehensive mapping of genetic co-expression among functionally defined cortico-striatal networks.

Specific striatal sub-regions functionally couple to dissociable cerebral networks^9,21^. Structurally, cortical projections extend from anterior to posterior striatum in longitudinal zones^23^, with ventromedial striatum receiving projections primarily from limbic (ventromedial and orbital frontal) cortex, central striatum from association cortex, and dorsolateral striatum from sensory-motor-related areas^11,12,19,22^. More complex projection patterns, including interdigitated and overlapping terminal fields, have also been observed^23^. In turn, the striatum sends ascending projections to cortex via the pallidal complex, substantia nigra, and thalamus, forming parallel but overlapping cortico-striatal circuits^11–13,23,24^. Given the stereotyped architecture of cortico-striatal circuits, a correspondingly stereotyped pattern of molecular co-expression may be present throughout the general population.

Synchronized patterns of gene expression could contribute to the formation and maintenance of functional brain networks^5^. By one view, network-associated gene expression across spatially distinct regions emerges as a function of common cell types^25^, potentially reflecting proximity during neurogenesis^26^. An alternate, but not mutually exclusive possibility is that convergent expression follows from shared profiles of activation or connectivity^27^, revealing the molecular machinery of network communication. Across cortex, variation in gene expression often takes the form of graded expression along a principal axis, for instance a rostral-caudal gradient^28,29^. Although spatial proximity captures a major aspect of molecular organization, converging evidence indicates that post-maturational gene co-expression tracks anatomical connectivity between neurons^30^ and among broader networks^31^. In humans, profiles of cortical gene expression recapitulate the topography of large-scale functional networks^5–8,32^, in part reflecting evolutionary innovations associated with long-range cortico-cortical projections^7^ and features of network connectivity^6,33^. Although independent lines of investigation have characterized gene expression in cortex^29^ and striatum^34,35^, the extent to which distributed cortico-striatal networks possess dissociable genetic signatures remains an open question.

Here we examine the association between cortico-striatal functional networks and gene expression in human post-mortem brain tissue. Observed patterns of correlated gene expression follow functional network topography of select cortico-striatal subnetworks. A limbic network (encompassing the nucleus accumbens (NAcc), orbital frontal cortex (OFC), and temporal pole) displays the greatest number of genes with consistent, network-associated cortico-striatal expression, including the inhibitory interneuron marker somatostatin (*SST*) and transcripts encoding two SST receptors (*SSTR1* and *SSTR2*). Common patterns of expression are also observed in the somato/motor cortico-striatal network (encompassing dorsal putamen and motor, auditory, and sensory cortices), including expression of the interneuron marker parvalbumin (*PVALB*). Observed network-associated gene expression is consistent across independent human datasets and evolutionarily conserved in non-human primates. These results suggest that functionally coupled aspects of cortex and striatum recapitulate spatial profiles of gene expression and provide insight into common molecular associates of cortico-striatal function across human and non-human primates.

## Results

### Topography of cortico-cortical genetic correlations

**Fig. 1:**
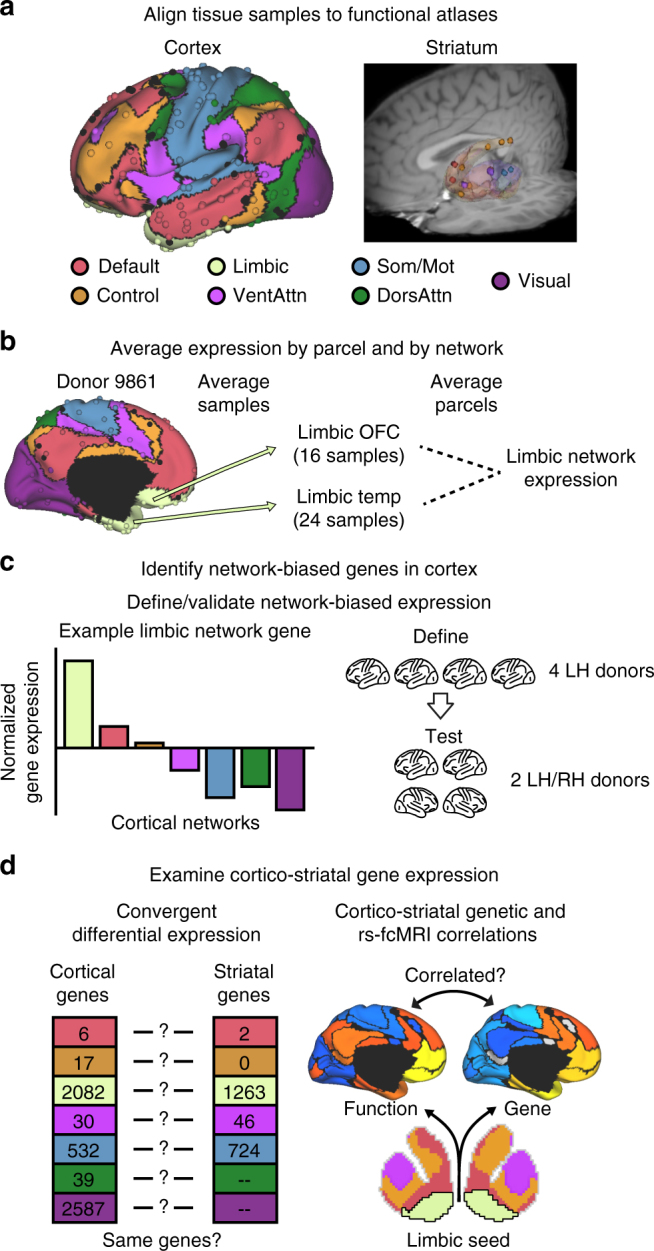
Characterizing the correspondence between cortico-striatal functional architecture and gene expression. **a** Individual tissue samples from the Allen Human Brain Atlas were aligned to the cortical functional connectivity atlas of Yeo et al.^36^ and the striatal atlas of Choi et al.^9^. Samples were grouped into default, frontoparietal control, limbic, ventral attention, somato/motor, dorsal attention, and visual networks. **b** For each individual donor, gene expression was averaged according to functional parcel, then by overall network, resulting in a single expression vector for each network in each donor. **c** Differential expression analyses revealed genes with biased expression across cortical networks. For instance, genes expressed most in tissue samples falling within limbic (cream) network regions relative to all others. Network-biased genes were initially identified in cortex of the four left hemisphere donors and cortico-cortical correlations were examined in the two bi-hemispheric donors. **d** Network-biased genes were re-defined in the cortex of all six available AHBA donors and were cross-referenced to network-biased genes in the corresponding region of the striatum. The genetic and resting-state functional correlation between each striatal sub-region and each cortical parcel is then calculated and compared

Publicly available human postmortem gene-expression data (*n* = 6) were obtained from the Allen Human Brain Atlas (AHBA; http://human.brain-map.org; Supplementary Table 1)^29,32^. The Montreal Neurological Institute (MNI) coordinates of each tissue sample were referenced to 51 approximately symmetric cortical regions from the Yeo et al.^36^ seven-network parcellation, including visual, somato/motor, dorsal attention, ventral attention, frontoparietal control, default, and limbic networks (Fig. 1a and Supplementary Tables 2 and 3). Genes expressed more in one network relative to all others were identified using differential expression analyses (Fig. 1b–c). Conventional criteria for differential expression includes a statistical threshold and a minimum ratio of expression in target relative to comparison samples (i.e., fold change)^32^. As gene expression is relatively homogenous across the cortex^29,32^, an arbitrary fold change threshold could mask subtle, yet biologically meaningful, variations in expression^37^. Accordingly, we adopted a statistical threshold to account for multiple comparisons (false discovery rate (FDR) corrected *q* ≤ 0.01) and used independent data (e.g. Brainspan and Genotype-Tissue Expression (GTEx) data) to assess population-level stability. Donors were initially divided into two groups to characterize cortical patterns of genetic correlation. Differentially expressed genes were identified in a discovery sample (*n* = 4 left hemisphere donors) and cortico-cortical gene co-expression was tested with independent data (*n* = 2 bi-hemispheric donors).

Analyses revealed 2664 unique genes with differential cortical network expression in the four discovery donors (*q* ≤ 0.01; i.e., genes with higher expression within a given network, relative to other networks). The majority of genes were preferentially expressed in limbic (count = 1326) and visual (count = 1264) networks, with the remainder in somato/motor (count = 169), ventral attention (count = 1), dorsal attention (count = 8), control (count = 3), and default (count = 0) networks.

**Fig. 2:**
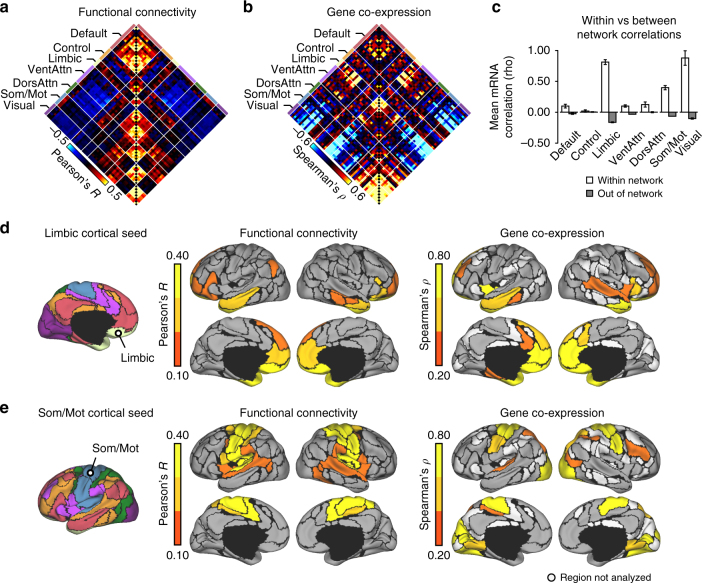
Anterior and posterior genetic gradients in the cortex. **a** Correlation matrix shows fcMRI based coupling for 59 cortical regions (*N* = 1000) from the 17-network parcellation of Yeo et al.^36^, corresponding to cortical areas containing tissue samples from both bi-hemispheric AHBA donors. Regions are arranged such that those belonging to the same functional network are grouped together. Functional network correlations reveal both positive (red) and negative (blue) associations. **b** Correlation matrix shows the coupling of gene expression across the cerebral cortex, averaged across the two bi-hemispheric AHBA donors. A total of 2664 genes were examined, defined based on differential expression across cortical networks in independent data from 4 left-hemisphere AHBA donors (*q* ≤ 0.01). **c** Bar graphs display the gene expression similarity (region-to-region correlation) for parcels within, and out of, the corresponding network territories. Data points reflect the average within and between network correlation values for each bihemispheric donor. Error bars reflect ± 1 SEM. **d** The average functional and genetic correlation profiles of bilateral limbic OFC seed regions and **e** bilateral dorsal somato/motor seed regions, displayed across medial and lateral surface representations of the 17-network cortical parcels from Yeo et al.^36^. DorsAttn, dorsal attention; Som/Mot, somato/motor; VentAttn, ventral attention and salience

Before examining cortico-striatal patterns of gene co-expression, we characterized the topography of cortico-cortical genetic correlations. Analogous to correlating resting-state blood-oxygen level dependent (BOLD) time series, mean-normalized expression of the 2664 differentially expressed genes was correlated across each cortical parcel. Differentially expressed genes were defined according to the 7-network cortical atlas, but region-to-region correlations were calculated using a finer, 17-network parcellation to provide more spatial precision (Supplementary Fig. 1). Consistent with prior reports^5–8,32^, region-to-region correlations of gene expression broadly recapitulated aspects of cortical functional architecture (Fig. 2a–c). Across all region-to-region pairs, genetic correlations associated with functional correlations (*r* = 0.28, *p* ≤ 0.001). Limbic network regions displayed strong cross-network correlations to anterior aspects of the default and ventral attention networks (Fig. 2b). To illustrate this general anterior weighted pattern of gene co-expression, we plotted the averaged correlation profile of bilateral limbic OFC regions on cortical surface (Fig. 2d). Further, somato/motor and visual network regions displayed expression profiles that were consistently distinct from those of limbic and association network regions (Fig. 2b), a profile that recapitulates prior analyses of circumscribed gene sets^7^. Figure 2e displays the averaged cortical correlation profile of bilateral dorsal somato/motor seed regions, which strongly correlated with regions in visual and dorsal parietal cortex. These examples serve to describe two dominant cortical co-expression patterns that emerge from our particular data-derived gene set.

To maximize the stability of our cortical gene set before assessing striatal expression, we recomputed network specific expression profiles in the available data from all six-donors (limbic = 2082, visual = 2587, somato/motor = 532, ventral attention = 30, dorsal attention = 39, control = 17, and default = 6). The addition of the two bi-hemispheric donors resulted in a ~ 84% increase in identified network-associated genes (count = 4912 unique genes; Supplementary Data 1), likely to be due to increased power given the small number of available donors.

### Convergent expression across cortico-striatal networks

**Fig. 3:**
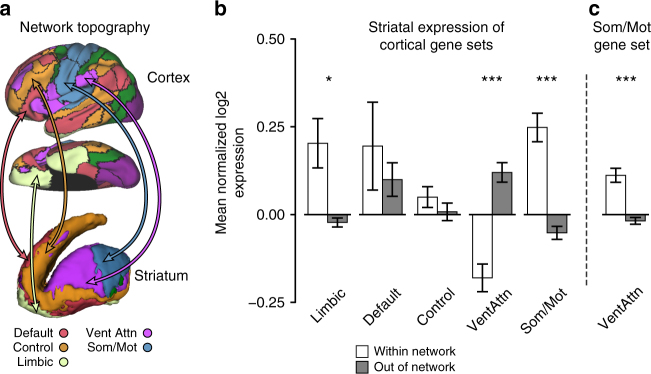
Network-associated genes defined in the cerebral cortex show network-biased expression in the limbic and somato/motor striatum. **a** Schematic illustrating the network architecture of cortex (above/middle) and striatum (below), displayed on the lateral and ventral surfaces of the left hemisphere and striatum. **b** Bar graph displays the gene expression (mean-normalized log2) of network-biased genes defined from cortex within, and out of, the corresponding striatal network territories using all six AHBA donors. Striatal tissue samples were not available for the dorsal attention and visual networks. Expression was greater for out of network, than within network, genes for ventral attention striatum. Error bars reflect ± 1 SEM. **c** Ventral attention aspects of striatum, relative to the rest of the striatum, displayed significantly greater expression of the somato/motor cortical gene set. *p ≤ 0.05, **p ≤ 0.01, ***p ≤ 0.005

Correspondence of gene expression across cortex and striatum was assessed by aligning AHBA striatal samples to the 7-network striatal atlas of Choi et al.^9^ (Fig. 3a, Supplementary Fig. 2, and Supplementary Tables 4 and 5). Dorsal attention and visual networks are minimally represented in the striatal functional atlas and largely did not overlap with striatal AHBA samples. They were therefore excluded from subsequent analyses. Overall, within-network expression (e.g., the 2082 limbic-biased cortical genes in limbic ventral striatum) was higher than out-of-network (e.g., limbic cortical genes in somato/motor striatum) expression for cortically defined network gene sets (*F*_1,5_ = 15.1, *p* ≤ 0.05; Within: *M* = 0.10, SE = 0.02; Between: *M* = 0.03, SE = 0.02; Fig. 3b). Post-hoc tests revealed that significant within-network expression was driven by limbic (*F*_1,5_ = 8.68, *p* ≤ 0.05) and somato/motor (*F*_1,5_ = 27.41, *p* ≤ 0.005) networks. Within-network expression was not observed in regions of striatum corresponding to default (*F*_1,5_ = 0.41, *p* = 0.55), control (*F*_1,5_ = 0.96, *p* = 0.37), and ventral attention networks (*F*_1,5_ = 27.98, *p* ≤ 0.005; greater between- than within-network expression). The significant negative relationship observed in ventral attention (violet) striatum is due to its strong genetic convergence with the somato/motor (blue) network, as the somato/motor cortical gene set displayed increased expression within ventral attention striatum relative to all other striatal parcels (*F*_1,5_ = 29.63, *p* ≤ 0.005; Fig. 3c).

We next derived gene sets displaying network-specific expression in the striatum (limbic = 1263, somato/motor = 724, ventral attention = 46, default = 2, and control = 0). For limbic and somato/motor cortico-striatal networks, we observed significant overlap of differentially expressed genes within both the cortex and striatum (limbic overlap = 505, somato/motor overlap = 108, and hypergeometric ps ≤ 0.001). That is, 505 genes were positively differentially expressed within both limbic striatum and limbic cortex. Likewise, 108 genes were positively differentially expressed within both somato/motor striatum and somato/motor cortex relative to all other networks. Parallel enrichment patterns were not evident within other functional networks (overlaps = 0). The putative biological functions of the 505 AHBA limbic cortico-striatal genes were characterized by comparing against annotated gene sets through ToppGene^38^, using backgrounds of all genes within a given annotation category (e.g., GO Biological Process). Analysis revealed enrichment for multiple receptor signaling mechanisms, including GABA-A receptor complex (GABA-A receptor complex, *q* ≤ 0.05), chloride channel complex (GO:0034707, *q* ≤ 0.05), neuropeptide signaling pathway (GO:0007218, *q* ≤ 0.05), and anterograde trans-synaptic signaling (GO:0098916, *q* ≤ 0.05; Table 1 and see Supplementary Data 1 for full enrichment table). Table 1 displays a subset of gene enrichment terms and serves to highlight specific genes contained within the limbic gene set. Limbic cortico-striatal biased genes also showed enrichment for genes associated with autism, a disorder characterized by abnormalities within cortico-striatal circuits^39^ (Table 1). The limbic network-biased gene set significantly overlapped with the genes theorized to support synchronous activity in cortico-cortical networks (overlap = 16, *p* ≤ 0.001)^5^ and which are linked to the amplitude of low frequency fluctuations in the default network (overlap = 10, *p* ≤ 0.005)^8^. The somato/motor network-associated gene set was enriched for genes preferentially expressed in oligodendrocytes (Supplementary Data 1, *q* ≤ 0.001). Consistent with recent reports of gene expression in cortex and downstream striatal projection sites in rodents^40,41^, *SST* and related receptors (*SSTR1*, *SSTR2*) were enriched in the distributed limbic network, whereas *PVALB* was preferentially expressed within the somato/motor network.

**Table 1:** Limbic network-biased gene set enrichment

	*p*	*q*	Genes
*Cellular component*			
Chloride channel complex	1.78E – 04	8.44E – 03	*GABRA3, GABRA5, GABRB1, GABRB3, GABRG1, GLRA2, GLRA3*
Cell projection part	1.29E – 05	2.38E – 03	*SLC26A4, PDYN, GPRIN1, FKBP1A, AKAP5, MAPK1, ATP2B4, GAP43, LAMP5, GLI1*
Synapse	6.58E – 05	4.00E – 03	*SYNPR, RAB3C, DACT1, CADM1, SEMA4F, MYRIP, SCGN, PALMD, PTCH1, SHANK1*
GABA receptor complex	5.13E – 05	3.64E – 03	*GABRA3, GABRA5, GABRB1, GABRB3, GABRG1*
*Biological process*			
Neuropeptide signaling pathway	6.30E – 05	2.78E – 02	*PDYN, CARTPT, SSTR1, SSTR2, GLRA2, NPPA, GLRA3, OPRK1, OPRM1, TENM1*
Anterograde trans-synaptic signaling	1.21E – 05	1.01E – 02	*ASIC2, HRH1, AKAP5, HTR2C, CARTPT, STON2, SST, SYN2, BAIAP3, SHANK1, NPTX1, OXTR*
Central nervous system development	1.51E – 05	1.01E – 02	*CENPF, ASIC2, MECOM, FABP7, TTC8, MKL2, PGAP1, PCDH19, HPCAL4, AKAP5, KLHL1*
*Disease*			
Autistic disorder	5.18E – 06	9.22E – 03	*ASIC2, CSMD3, MKL2, CADM1, PCDH19, SCN3A,CELF6, MRC1, OXTR, MACROD2*

505 genes were differentially expressed in both limbic cortex and limbic striatum (*p* ≤ 0.001). Overlap with annotated gene sets were estimated using standard hypergeometric tests. *q*-values correspond to FDR Benjamini and Hochberg correction. Displayed are subsets of genes within each enrichment category

Illustrating that the limbic and somato/motor cortico-striatal convergence is not dependent upon arbitrary gene selection criteria, gene-wise log2 fold change was positively correlated across all available genes (count = 20,738) within limbic cortex and striatum (*r* = 0.35, *p* ≤ 0.001) and somato/motor cortex and striatum (*r* = 0.33, *p* ≤ 0.001). A negative fold change correlation was observed between limbic striatum and somato/motor cortex (*r* = −0.35, *p* ≤ 0.001), as well as between somato/motor striatum and limbic cortex (*r* = −0.35, *p* ≤ 0.001; Supplementary Fig. 3), highlighting that the observed associations are not obligated by a uniform property of striatal gene expression or broader profiles of expression in all brain tissue.

### Gene co-expression mirrors aspects of network architecture

**Fig. 4:**
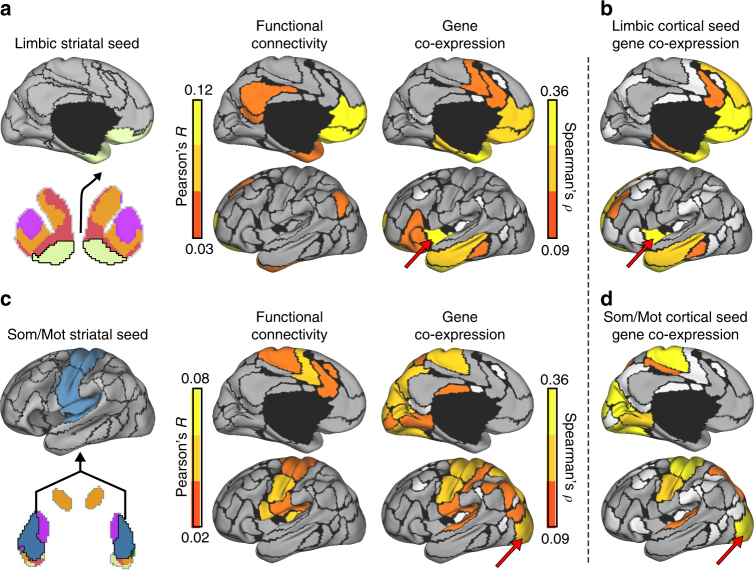
Functional and genetic cortical correlations of limbic and somato/motor striatal seeds. **a** The correspondence of fcMRI correlation patterns between the limbic region of striatum with cortex was calculated for the 73 bi-hemispheric cortical parcels using Pearson’s *r*. Corresponding gene expression correlations were calculated using Spearman’s *ρ*. Only left-hemisphere maps are displayed (see Supplementary Fig. 4 for unthresholded, bi-hemispheric maps). White denotes regions without samples from at least two donors. Limbic functional and genetic maps were correlated, *r* = 0.32, *p* ≤ 0.01. **b** The cortical gene expression correlations of bilateral limbic OFC (as in Fig. 1d) were associated with the genetic correlation profile of limbic striatum, *r* = 0.88, *p* ≤ 0.001. Red arrows highlight an example region where cortico-striatal genetic correlations differ from functional connectivity, but aligns to cortico-cortical genetic correlations of the limbic OFC. **c** The functional and genetic correlations of the somato/motor striatal seed region. **d** The cortical gene expression correlations of bilateral dorsal somato/motor cortex (from Fig. 1e) followed the genetic correlation profile of somato/motor striatum, *r* = 0.92, *p* ≤ 0.001. Red arrows highlight an example region where cortico-striatal genetic correlations differ from functional connectivity, but aligns to cortico-cortical genetic correlations of the dorsal somato/motor cortex

The previous analyses identify genes that are differentially expressed across cortico-striatal networks. However, gene co-expression may associate with functional connectivity in a manner not fully captured through differential expression analyses. We examined the extent to which region-to-region gene co-expression recapitulates cortico-striatal functional network architecture. Using the cortically defined network genes, cortico-striatal gene expression correlations were estimated across cortical parcels containing samples from 2 or more donors (73/114 cortical parcels). Because of marked differences in global expression profiles between cortex and subcortex^29^, expression values were mean-normalized separately for cortex and striatum to reveal parallel patterns of relative expression. Correlations were estimated with Spearman’s correlation for microarray data (log2 mean-normalized) and Pearson’s correlation for fcMRI data (*n* = 1000)^9,42^. Suggesting a shared network structure, a correspondence was observed across the genetic and functional correlations linking striatum to cortex for default (*r* = 0.44, *p* ≤ 0.001; Supplementary Fig. 4) and limbic networks (*r* = 0.32, *p* ≤ 0.01; Fig. 4a). Although the insula was not strongly functionally correlated to ventral striatum, the observed genetic correlation linking limbic striatum and the insula is consistent with descending cortico-striatal projections from the insula to NAcc and ventral caudate^10,43^. The profile of cortical genetic correlations for limbic striatum and the adjacent default striatum were highly convergent (*r* = 0.62, *p* ≤ 0.001), indicating that coexpression patterns between limbic striatum and cortex reflect gradual expression gradients, rather than differential expression between NAcc and the rest of the striatum.

**Fig. 5:**
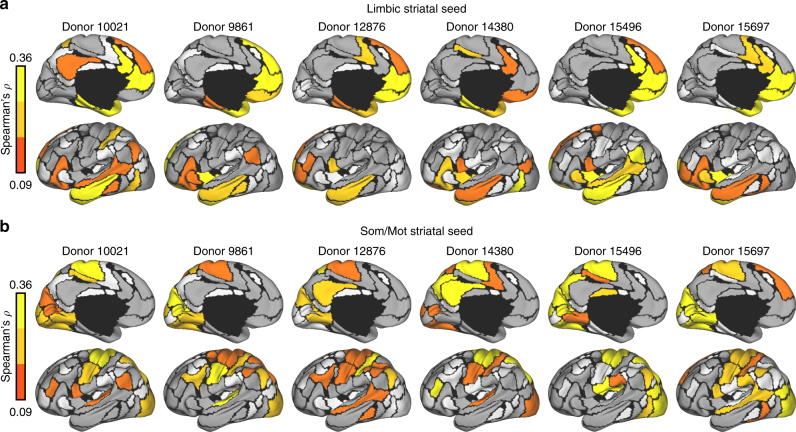
Cortico-striatal genetic correlations are consistent within individual donors. Genes exhibiting differential expression across cortical networks in all six AHBA donors were examined (count = 4912). For each donor, gene expression in the **a** limbic and **b** somato/motor striatal regions were correlated to each cortical region for which data was available. White denotes regions for which no data were available. Grey denotes Spearman’s correlations < 0.09

The overall correlation between functional connectivity and gene co-expression was not significant for somato/motor (*r* = *−* 0.08, *p* = 0.49; Fig. 4b), control (*r* = *−* 0.16, *p* = 0.17), and ventral attention (*r* = *−* 0.13, *p* = 0.27) striatal regions (Supplementary Fig. 4). However, somato/motor striatum was strongly positively correlated to each somato/motor cortical parcel (*M* = 0.19, SE = 0.04; Fig. 4c). Ventral attention and somato/motor striatal regions, which both occupy posterior putamen, showed overlapping genetic correlation patterns across cortex (*r* = 0.93, *p* ≤ 0.001), suggesting that those striatal regions may not possess dissociable molecular signatures (see Supplementary Fig. 4 for all unthresholded maps). Patterns of gene co-expression of limbic and somato/motor striatal seeds strongly recapitulated the respective cortical correlation maps derived from limbic OFC (*r* = 0.88, *p* ≤ 0.001; Fig. 4b) and somato/motor cortex (*r* = 0.92, *p* ≤ 0.001) seed regions (Fig. 4d), indicating a convergent genetic profile among these aspects of cortico-striatal networks. For instance, somato/motor striatum correlates strongly with visual cortex (Fig. 4c, red arrow), which is consistent with the genetic correlation profile of the somato/motor cortical seed (Fig. 4d, red arrow). Further highlighting that the network structure of cortico-striatal gene expression reflects a stable individual-level phenomenon, observed patterns of messenger RNA co-variation were highly consistent across individual donors (Fig. 5).

### Differential expression replicates in independent data

**Fig. 6:**
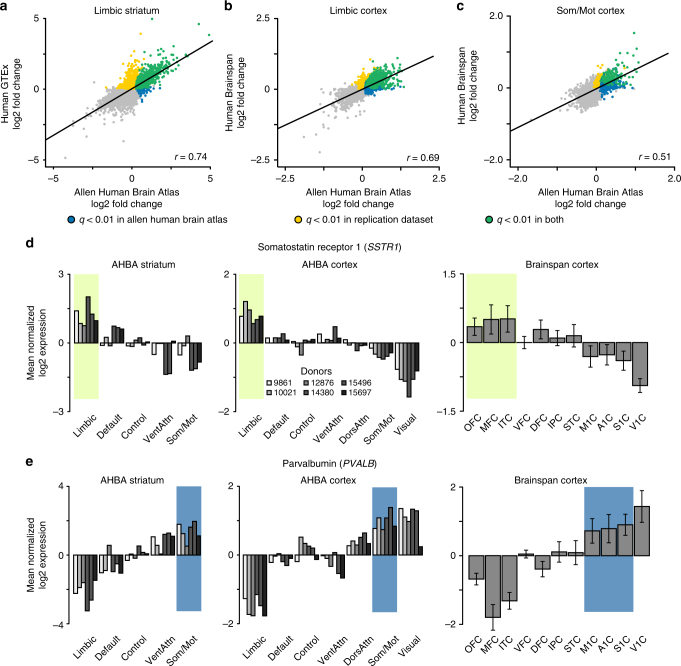
Differential expression across cortical and striatal networks is consistent within independent human datasets. **a** RNAseq data from 111 putamen, 130 NAcc, and 144 caudate tissue samples were obtained from the Genotype-Tissue Expression (GTEx) project. GTEx samples from the NAcc correspond to the limbic aspect of striatum in the AHBA dataset. Across all genes, differential expression (i.e., log2 fold change) in GTEx NAcc, relative to caudate and putamen, co-varied with differential expression in AHBA limbic striatum (*r* = 0.74, *p* ≤ 0.001). **b** RNAseq data for 8 adult donors sampling 11 cortical brain regions were obtained from the Brainspan atlas. Across all genes, fold change in Brainspan limbic cortex (i.e., OFC, ACC, ITC) correlated with fold change in the AHBA limbic cortex (*r* = 0.69, *p* ≤ 0.001). **c** Gene-wise fold change in Brainspan somato/motor cortex (i.e., M1C, S1C, A1C) correlated with fold change in the AHBA somato/motor cortex, *r* = 0.51, *p* ≤ 0.001. Gray denotes genes that are not positively differentially expressed. **d** Somatostatin receptor 1 (*SSTR1*) was expressed most within limbic cortex and striatum of both AHBA and replication data. **e** Parvalbumin (*PVALB*) was expressed most within somato/motor cortex and striatum of both AHBA and replication data. Error bars reflect ± 1 SEM

To quantify whether network-associated expression may be common across the adult population, the stability of differential expression was examined in independent cortical (Brainspan Atlas of the Developing Human Brain)^44,45^ and striatal datasets (GTEx Project; http://www.gtexportal.org)^46^. Precise spatial coordinates of GTEx and Brainspan tissue samples were not available. Anatomical labels were used to identify samples within structural territories that broadly correspond to functionally defined limbic striatum, limbic cortex, and somato/motor cortex^36^. Gene-wise fold change for the GTEx NAcc (*n* = 165 donors), relative to the caudate and putamen, was strongly correlated to gene-wise fold change in the AHBA limbic striatum, relative to non-limbic striatal regions (*r* = 0.74, *p* ≤ 0.001; Fig. 6a), suggesting a stereotyped expression architecture across the adult population.

Brainspan RNA sequencing (RNAseq) data were used to estimate the consistency of gene expression within limbic and somato/motor aspects of cortex (*n* = 8 donors). Samples were available from 11 cortical regions, including three (orbitofrontal cortex (OFC), anterior cingulate cortex (ACC), and inferolateral temporal cortex (ITC)) territories broadly corresponding to the limbic cortical network^36^. Differential expression in OFC, ACC, and ITC was calculated relative to the eight other sampled cortical regions and was significantly correlated to fold change within AHBA limbic cortex (*r* = 0.69, *p* ≤ 0.001; Fig. 6b). Likewise, expression of somato/motor cortex was calculated using samples from primary motor cortex (M1C), primary sensory cortex (S1C), and primary auditory cortex (A1C). Gene expression within the somato/motor network was consistent across the Brainspan and AHBA datasets (*r* = 0.51, *p* ≤ 0.001; Fig. 6c), further supporting the reliability of observed cortical expression profiles. Preferential expression of *SST, SSTR1*, and *SSTR2* within limbic (cream) regions and *PVALB* within somato/motor (blue) regions, respectively, was consistent in independent replication data (Fig. 6d, e).

### Conservation of gene expression in non-human primates

**Fig. 7:**
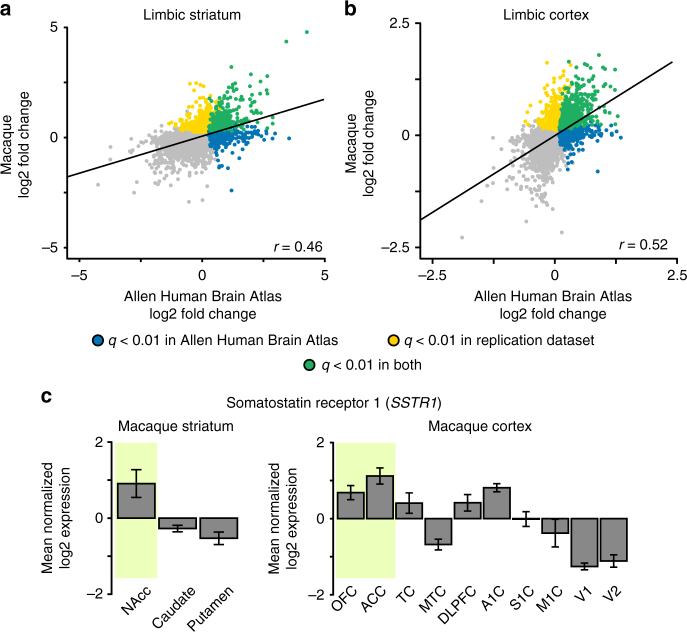
Differential expression in limbic cortical and striatal networks is conserved in non-human primates. **a** Microarray data from the NAcc, putamen, and caudate of six adolescent and young adult macaque primates were obtained from the NIH Blueprint Non-Human Primate atlas. Gene-wise differential expression (i.e., log2 fold change) in Blueprint non-human primate NAcc, relative to caudate and putamen, was positively correlated to differential expression in human AHBA limbic striatum, *r* = 0.46, *p* ≤ 0.001). **b** Cortical microarray data for six adult macaque primates sampling ten cortical brain regions were obtained from the study by Bernard et al.^28^. Gene-wise log2 fold change in primate limbic cortex (i.e., OFC, ACC) was positively correlated to fold change in the AHBA limbic cortical region, *r* = 0.52, *p* ≤ 0.001). **c** Somatostatin receptor 1 (*SSTR1*) was expressed most within limbic cortex and striatum of the macaque. Error bars reflect ± 1 SEM

Aspects of cortico-striatal anatomy and function are preserved across humans and non-human primates^10^. To test whether network-biased gene expression is consistent in primates, we analyzed gene expression in rhesus macaque monkeys (*Macaca mulatta)* from the NIH Blueprint Non-Human Primate atlas (http://www.blueprintnhpatlas.org/)^47^. Microarray data of samples of adolescent (*n* = 3) and young adult (*n* = 3) macaque monkeys were analyzed, using the high-confidence human-macaque gene homologs identified by Bakken et al.^47^ (count = 10,616). Macaque NAcc fold change, relative to the caudate and putamen, significantly correlated to fold change of AHBA limbic striatum (*r* = 0.46, *p* ≤ 0.001; Fig. 7a), as well as that of GTEx NAcc (*r* = 0.40, *p* ≤ 0.001). Homologous expression within primate somato/motor striatum could not be examined given that the somato/motor striatal parcel does not align to anatomical divisions between the NAcc, caudate, and putamen.

Cross-species consistency of differential expression in cortex was tested using microarray data from adult macaque monkeys (*n* = 6) sampled across 10 cortical regions^28^. Differential expression in macaque limbic cortex (OFC and ACC samples) relative to the eight other regions revealed consistent expression patterns with those found in the limbic-cortex in humans (Fig. 7b; AHBA: *r* = 0.52, *p* ≤ 0.001; Brainspan: *r* = 0.49, *p* ≤ 0.001). Analysis of somato/motor cortex revealed a muted, but significant pattern of correlated differential expression between homologous cortical regions in macaque and AHBA samples (*r* = 0.13, *p* ≤ 0.001), which was not evident in the adult human Brainspan data (*r* = −0.07, *p* ≤ 0.001). As with the human limbic network, *SST* (striatum, *q* ≤ 0.005; cortex, *q* ≤ 0.001) and *SSTR1* (striatum, *q* ≤ 0.001; cortex, *q* ≤ 0.001) were expressed significantly more within primate limbic cortex (Fig. 7c). Likewise, *PVALB* was preferentially expressed in primate somato/motor cortex (*q* ≤ 0.05).

### Limbic genes are expressed across cell types and layers

**Fig. 8:**
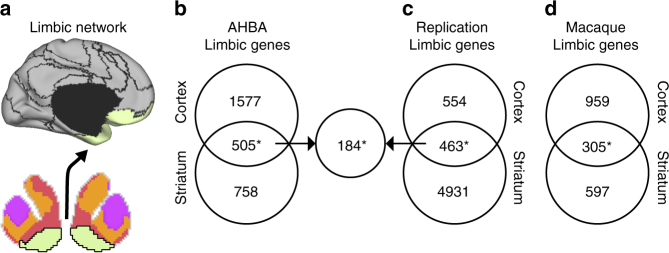
Limbic network-biased genes are consistent in independent human and non-human primate datasets. **a** Schematic illustrating the limbic cortico-striatal network displayed on a bi-hemispheric cross-section of the striatum and the lateral surface of the left hemisphere. **b** Among the AHBA data, 505 genes displayed overlapping positive differential expression for both limbic striatum and limbic cortex (hypergeometric *p* ≤ 0.001). **c** Among the GTEx and Brainspan replication data, 463 genes displayed overlapping positive differential expression for limbic striatum and limbic cortex, 184 of which overlapped with the AHBA limbic network gene set (ps ≤ 0.001). **d** In macaques, 305 genes displayed overlapping positive differential expression for limbic striatum and limbic cortex, 93 of which overlapped with the AHBA limbic network gene set (ps ≤ 0.001). *p ≤ 0.001

Across all datasets, an overlapping set of genes displayed increased expression within limbic cortico-striatal network (Fig. 8). Consistent with our analysis of the AHBA data, we identified genes that were positively differentially expressed in GTEx NAcc relative to the caudate and putamen (*q* ≤ 0.01; count = 5394), as well as in Brainspan limbic cortex relative to all other cortical samples (*q* ≤ 0.01; count = 1017). Of these, a common set of 463 genes were significantly differentially expressed in both limbic cortex and limbic striatum of the replication data, which exceeds the overlap that would be expected by chance (hypergeometric *p* ≤ 0.001). Thirty-nine percent (count = 184) of these genes overlapped with the 505 limbic network-biased genes identified in the AHBA dataset (Fig. 8; *p* ≤ 0.001). Due to an inability to localize samples to somato/motor striatum in GTEx data, we are unable to draw conclusions regarding the stability of cortico-striatal expression within this network. Nevertheless, these results indicate that profiles of limbic network gene expression are highly stereotyped across the population. A set of 305 genes were differentially expressed in both the macaque limbic cortex and limbic striatum (*p* ≤ 0.001). Ninety-three of these genes overlapped with the limbic network-associated genes identified in the AHBA donors and 90 overlapped with the GTEx/Brainspan limbic-network genes (Fig. 8; *p* ≤ 0.001). These data suggest that molecular patterns of gene expression are evolutionarily conserved within limbic aspects of cortico-striatal circuitry, and that non-human primates may provide an important comparator for the study of genetically mediated aspects human cortico-striatal development and function.

Single-cell expression data from mouse cortex^48^ was used to test whether the limbic network-biased gene set was enriched for specific cell types (i.e., astrocytes, neurons, oligodendrocyte precursor cells, newly formed oligodendrocytes, myelinating oligodendrocytes, microglia, and endothelial cells). Of AHBA limbic network biased genes with identifiable mouse homologs (count = 388), 130 (34%) displayed any form of cellular enrichment, operationalized as 1.5 log2 fold change in the most expressed cell type relative the second most expressed cell type. For genes exhibiting preferential cell type expression, the majority were enriched for neurons (count = 69), with the remainder enriched for astrocytes (count = 20), microglia (count = 16), endothelial cells (count = 14), oligodendrocyte precursor cells (count = 10), and newly formed oligodendrocytes (count = 1; Supplementary Data 1). These data indicate that convergent patterns of mRNA expression emerge as a function of expression within multiple cell types across both striatum and cortex (see also Supplementary Fig. 5). The majority of AHBA limbic-associated genes did not display laminar-specific expression in macaque OFC and ACC, indicating that cortico-striatal transcriptional correspondence in the limbic circuit is not driven by genes expressed in the striatal-targeting cortical projection neurons that primarily cluster in deeper layers 5/6 (Supplementary Fig. 6)^13^.

## Discussion

In this study we demonstrate that molecular profiles, defined at the level of cortex, display consistent expression within functionally coupled aspects of striatum. Patterns of cortico-striatal gene expression in limbic and somato/motor networks were highly stable across individuals, replicated in independent datasets, and were evolutionally conserved in non-human primates. These data suggest that sites of correspondence between gene expression and functional brain architecture are stereotyped, supporting the use of circuit-level transcriptional analysis to discover molecular markers of network function in health and disease. Our analyses are consistent with recent evidence in rodents that limbic and somato/motor processing channels are differentially associated with SST and PVALB neurons^40,41^. Coupling of inter-regional gene expression may emerge, in part, via anatomical connectivity^30,31^. We extend this concept of conserved transcriptional patterning to functional architecture, demonstrating network-biased expression in both the cortical and striatal aspects of limbic and somato/motor networks.

For some genes, consistent patterning across functionally coupled regions may indicate their relevance for circuit integrity and associated domains of behavior and disease. In line with this speculation, our results converge with evidence for dissociable molecular signatures of limbic and somato/motor pathways in striatum, in part driven by the differential presence of SST and PVALB interneurons^49^. In addition to the previously noted preferential expression of *SST* related receptors (*SSTR1, SSTR2*) in limbic cortex and striatum, analyses revealed enrichment of neuropeptide signaling genes known to co-localize with SST neurons (e.g., *PDYN*)^50^ and with limbic-associated striatal patch regions (*OPRM1*, *OPRK1*)^51^. These results provide evidence that *SST* is preferentially expressed within human limbic striatum as well associated cortical input structures. Taken together, these data point to potential molecular associates of cortico-striatal limbic function and related affective disorders. *SST* is reduced within the subgenual ACC of depressed patients^52^. In rodents, knockdown of *SST* expression produces a depressive phenotype^53^, whereas the disinhibition of SST neurons produces anti-depressant like effects^54^. Further, meta-analyses reveal reduced cortical thickness in depressed patients within medial prefrontal and temporal regions^55^ that show strong limbic cortico-striatal correlations within the current study. Our analyses additionally revealed genes implicated in ventral-striatal mediated behaviors, including *OXT, GABRA3*, *GABRA5*, *GABRB1*, and *GABRB3*, which have previously been linked to mesolimbic reward circuitry and risk for associated disorders^56–58^. Of note, 14% of the 505 AHBA limbic cortico-striatal genes have no established gene ontology annotation (count = 71), supporting the use of gene co-expression analyses as a tool for nominating genetic markers of behavior and mental illness.

Within the somato/motor cortico-striatal network, our analyses revealed the preferential expression of *PVALB* and myelin-associated transcripts (e.g., *MOBP*, *MYLK*, *GJB1*, and *MITF*). Rodent immunocytochemical studies suggest *PVALB* is preferentially expressed within basal ganglia structures that are innervated by the somato/motor dorsolateral striatum^59^. More recent work confirms that rodent *PVALB* expressing neurons are the primary source of basal ganglia innervation of motor thalamus^41^. The coexpression of *PVALB* with oligodendrocyte-related transcripts may be driven by the presence of myelinated projection neurons or due to preferential myelination of PVALB interneurons^60^. Together, these spatial patterns of expression indicate the relative increased presence of parvalbumin containing cells within cortico-striato-thalamic motor networks.

The structure and function of cortico-striatal circuitry is conserved across primates^10^. We provide evidence that molecular machinery of limbic and somato/motor regions are similarly conserved. Non-human primates and rodents may serve as important comparators for understanding the functional consequences of gene co-expression for health and disease, particularly when temporally frequent sampling of pre- and post-natal periods and dense anatomical coverage are required^61^. Approximately 9% of genes have dissociable expression trajectories in rhesus monkeys and humans, including genes with delayed peak expression solely in human cortex^47^. Here as well, we identified features of transcription that varied in their conservation across human and non-human primates. Differences in gene expression across species will be critical for understanding unique features of brain evolution, such as the late developing association networks and human-specific pathology.

Our analyses of gene expression and functional-connectivity MRI are not without limitations. For instance, there is substantial evidence for individual differences in network organization that are non-uniformly distributed across the cortex^62^, with increased population-level variability in association relative to unimodal cortices^63,64^. Although speculative, the muted function-expression relationships observed in association cortex may be partly explained by high inter-individual functional variability within these regions. The present results do not suggest the presence of global or network-general correspondence between cortico-striatal gene expression and functional connectivity, as differences are readily apparent. For instance, the default seed of the striatum does not show genetic correlations with the posterior cingulate cortex (Supplementary Fig. 4b) and the somato/motor striatal seed shows strong correlations to visual as well as somato/motor cortical areas (Fig. 4c). In addition, our analyses reveal preferential patterns of expression within a network and should not be taken to preclude their potential importance across other regions. Rather, increased expression of a gene among functionally coupled territories may reveal a preferential functional role within the associated network.

It is possible that tissue homogenates in both cortex and striatum capture components of the same long-range neurons. Although local translation of mRNA can occur away from the cell nucleus^65^, we do not expect gene expression in distal components of a cell (e.g., synapse) to be the same as in the cell body. Nonetheless, we partially address this possibility by examining laminar expression patterns in cortex (Supplementary Fig. 6), revealing that limbic network-biased genes do not localize to deeper layers of OFC and anterior cingulate, where ventral striatum-projection neurons cluster^13^. However, future studies should examine the degree to which correspondence of cortico-striatal gene expression and network organization is driven by GABAergic interneurons, particularly given evidence that PVALB and SST cortico-striatal projections differentially modulate behavior^66^. Although our analyses prioritize a specific tractable set of genes for future study, it is not currently feasible to link individualized estimates of network function and gene expression in humans, although methods for transcriptome inference are emerging^67^. Exploring the nature of such relationships will be a worthwhile avenue of research, likely requiring the use of animal models and large-scale collaborative efforts in humans, encompassing in vivo estimates of brain function and structural genetic variation.

Here, using the Allen Institute, Brainspan, GTEx, and NIH Blueprint brain transcriptional atlases, we demonstrate that spatial patterns of gene expression recapitulate limbic and somato/motor cortico-striatal functional networks. The resulting transcriptional profiles provide novel gene targets for future research on the development and maintenance of cortico-striatal circuitry, and associated behaviors.

## Methods

### Allen Human Brain Atlas

Publicly available gene expression data from six human postmortem donors (*n* = 1 female), aged 24–57 years of age (*M* = 42.5, SD = 13.38), were obtained from the AHBA, downloaded after the updated microarray normalization pipeline implemented in March 2013 (http://human.brain-map.org)^29^. Relevant demographic information is also documented in Table S1. Analyses were conducted in accordance with guidelines set by the Yale University Human Subjects Committee.

### Brainspan Atlas

Eight adult donors between 18 and 40 years of age (*n* = 4 female, mean age = 28.00, SD = 8.85, range = 18–40yrs) from the Brainspan Atlas of the Developing Human Brain (http://www.brainspan.org/) were analyzed^44,45^. RNAseq reads per kilobase per million (RPKM) data from 11 cortical regions were examined: OFC, ACC, ITC, dorsolateral prefrontal cortex (DLPFC), ventrolateral prefrontal cortex, inferior parietal cortex, M1C, superior temporal cortex, primary visual cortex, A1C, primary somatosensory cortex (S1C). Information about data preprocessing and normalization is available on the Brainspan Atlas website (http://help.brain-map.org//display/devhumanbrain/Documentation).

### NIH Genotype-Tissue Experiment Project

RNAseq count data from 111 putamen, 130 NAcc, and 144 caudate tissue samples from 165 unique donors were obtained from the GTEx project (31.51% female)^46^. Age information was available in 10-year bins. The majority of donors were over 40 years of age (20–29 years, *n* = 6; 30–39 years, *n* = 2; 40–49 years, *n* = 20; 50–59 years, *n* = 53; 60–69 years, *n* = 75; 70–79 years, *n* = 9). GTEx data are openly available for download (GTEx Analysis V7; http://www.gtexportal.org/home/), and information about data normalization and processing is available online (http://www.gtexportal.org/home/documentationPage).

### Primate microarray data

Cortical microarray data from six adult non-human primates (50.00% female) analyzed by Bernard et al.^28^ was downloaded from the Gene Expression Omnibus website (https://www.ncbi.nlm.nih.gov/geo) under accession number GSE31613, which also provides information on data normalization. Samples from 10 cortical regions were examined: OFC, ACC, medial temporal lobe, DLPFC, temporal area, A1C, S1C, M1C, V1, and V2.

Striatal microarray data from an independent set of macaque primates was downloaded from the NIH Blueprint Non-Human Primate Atlas website (60.00% female; http://www.blueprintnhpatlas.org/)^47,68^, which contains the technical white paper describing data normalization procedures. Samples were available from macaque caudate, NAcc, and putamen, obtained from three adolescent (age = 12 months) and three young adult (age = 48 months) macaques.

### AHBA mRNA probe selection

Before tissue dissection, donor brains underwent anatomical MRI scanning and alignment to MNI space by the Allen Institute. Available samples (*n* = 4 left hemisphere only, *n* = 2 both left and right hemispheres) were prepared for microarray expression analyses by the Allen Institute via macro-dissection for cortical areas or laser dissection for subcortical regions^29^. For additional information on structural imaging data as well as microarray preprocessing and normalization procedures, refer to the AHBA technical white paper (help.brain-map.org/display/humanbrain/Documentation). A total of 58,692 microarray mRNA probes were available for each tissue sample. If three or more probes were present for a single gene, the probe with the maximum summed adjacency was retained, otherwise the probe with the highest mean expression was selected, using the CollapseRows function in R^69^. The resulting dataset contained 20,738 unique mRNA probes, providing transcriptional data of the cortex (*M* = 455.38, SD = 50.62 samples per hemisphere) and striatum (*M* = 20.50, SD = 3.56). Given known global transcriptional differences across subcortical and cortical regions, mean normalization of expression values was conducted separately for striatal and cortical tissues samples^29^. Primary analyses were completed using the R statistical software package (v. 3.1.0; http://www.r-project.org/).

### Aligning AHBA samples to functional network atlases

For cortical network assignment, a population functional atlas of human brain networks derived from 1000 healthy young adults was used^36,42^. The cortical atlas from Yeo et al.^36^ is publicly available for download (https://surfer.nmr.mgh.harvard.edu/fswiki/CorticalParcellation_Yeo2011). AHBA cortical tissue samples were referenced to both the 7- and 17-network parcellation, which respectively contain 51 and 114 spatially contiguous and approximately symmetric cortical parcels. Individual cortical surface parcellations were transformed from the Freesurfer surface^70–75^ to 1 mm^3^ MNI volumetric space (see Yeo et al.^36^ for details). Using AFNI^76^, single voxel (1 mm^3^) region of interest (ROI) was created at the MNI coordinate of each donor’s individual tissue samples and a functional network label was assigned if the ROI fell within a volumetric cortical parcel. Tables S2 and S3 summarize counts of cortical tissue network assignments for the 7- and 17-network solutions. Results were plotted on inflated surface representations of the cortex using Caret^77^.

The coordinates of each striatal sample were referenced to the volumetric 7-network functional striatal atlas of Choi et al.^9^ (http://surfer.nmr.mgh.harvard.edu/fswiki/StriatumParcellation_Choi2012). The 7-network atlas was selected to improve inter-subject comparisons by minimizing the number of striatal parcels with sparse or non-existent sampling (Supplementary Table 4) that occurred in the finer-grained 17-network atlas (Supplementary Table 5). Each AHBA striatal sample was assigned to a network if the 1 mm^3^ ROI overlapped with a striatal atlas network. For samples that did not initially overlap with the group atlas, the associated ROI was expanded to 2 mm^3^. If the 2 mm^3^ ROI overlapped with the functional atlas, the network with the most overlapping voxels in the ROI was assigned; otherwise the process was repeated for 3 mm^3^ ROI. A striatal sample was omitted from further analysis if the 3 mm^3^ ROI did not overlap with the striatal functional atlas. Supplementary Tables 4 and 5 shows summary counts of striatal network assignment. Few tissue samples overlapped with the visual or dorsal attention striatal functional parcels, which have sparse representation in the striatal functional atlas^9^, so they were omitted from analyses.

### Identifying differentially expressed genes across networks

AHBA gene expression of samples in the same spatially contiguous cortical region from the 7-network parcellation was averaged. Gene expression among regions belonging to the same network (i.e., default, limbic, etc.) were then averaged, resulting in a single expression value for each gene in each cortical network for each donor. Using the R package limma^78^, linear modeling identified gene-wise differential expression across the 7 networks by comparing the expression of a gene in one network (e.g., default) with all others (e.g., limbic, visual, etc). Given that gene expression across the cortical sheet is relatively homogenous^29^, a minimum fold change threshold was not applied to determine biologically meaningful differential expression. Rather, a statistical threshold of FDR Benjamini and Hochberg-corrected *q* ≤ 0.01 was adopted to account for multiple comparisons and we sought to replicate expression patterns in independent data (e.g., GTEx and Brainspan). Differential expression was calculated in the same manner in the striatum across limbic, default, control, ventral attention, and somato/motor striatal regions. For all differential expression analyses, residual donor effects were accounted for by using limma’s duplicateCorrelation tool^78^.

### Human differential expression replication analyses

For the cortical Brainspan data, 18,597 unique probes possessed matching Entrez IDs to the AHBA data and non-zero levels of expression across all samples. Duplicate probes were collapsed for each donor by selecting the probe with the maximum mean expression, using the CollapseRows function in R^69^. Additional normalization steps were not conducted. Three-dimensional coordinates were not available for Brainspan samples. Accordingly, network assignments of available samples were inferred based on their associated anatomical labels. The OFC, ACC, and ITC were assigned to the limbic network, and M1C, A1C, and S1C were assigned to the somato/motor network. Differential expression for Brainspan limbic and somato/motor cortex was calculated by comparing gene expression in the network of interest relative to all other cortical regions, controlling for sex and age. As raw count data are unavailable from the current Brainspan release, log2 RPKM values were used for differential expression analyses. The mean–variance relationship observed in RNAseq data was accounted for by fitting an intensity-dependent trend with the eBayes function (i.e., trend = TRUE).

For striatal NIH GTEx data, 15,786 probes possessed matching Entrez IDs to the AHBA data. Tissue samples were selected to reflect the quality control pipeline of the NIH GTEx, as described in the online white paper (V7; https://www.gtexportal.org/home/documentationPage). Briefly, RNAseq count data from each tissue were normalized using size factors implemented with DESeq2^79^. Genes were log-transformed and removed if they possessed fewer than 3 reads across all tissue samples or were not available in all three tissue types. Count data were normalized using the trimmed mean of M-values with the edgeR software^80^. When multiple counts were present for the same gene, the one with the highest mean expression was selected.

The functional striatal network assignment of caudate and putamen samples could not be inferred. However, all AHBA limbic striatal samples fell within the NAcc; thus, this region was selected to correspond to the limbic striatum. Differential expression for the GTEx limbic striatum (i.e., NAcc), relative to the caudate and putamen was calculated using voom^81^, controlling for age, sex, sequencing platform, and a set 15 covariates derived by NIH GTEx using the Probablistic Estimates of Expression Residuals method^82^.

### Primate differential expression analyses

A total of 10,616 genes unique high-confidence human to macaque homologs were examined, based on analyses by Bakken et al.^47^. In primate data from Bernard et al.^28^, the OFC and ACC were considered homologs to human limbic cortex, and M1C, A1C, and S1C were considered homologs to human somato/motor cortex. Microarray data from individual cortical lamina in the same region were averaged within each donor before analysis. Differential expression for limbic and somato/motor aspects of cortex was calculated using limma^78^ by comparing gene expression in the network of interest relative with all other cortical regions, controlling for sex. For striatal microarray data from the NIH NHP Atlas^47^, the NAcc was considered homologous to limbic striatum. Differential expression for limbic striatum (i.e., NAcc), relative to the caudate and putamen, was calculated using limma^78^, controlling for age.

### Cortical functional connectivity and gene co-expression

Cortico-cortical correlations of gene expression were examined in the two bi-hemispheric AHBA donors. Fifty-nine regions containing samples from both subjects were analyzed (Supplementary Fig. 1) across 2664 genes that showed differential patterns of gene expression across cortical networks in the 4 left-hemisphere donors. For each bi-hemispheric donor, log2 gene expression of cortical samples was mean-normalized and then averaged within regional parcels. Spearman’s *ρ* was used to calculate cortical region-to-region correlations separately for each donor. The correlation matrices for each of the bi-hemispheric donors were Fisher transformed and then averaged. Cortico-cortical correlations of resting-state BOLD time series were estimated among the same 59 cortical regions, with data from 1000 healthy individuals^36,42^. Full information on the pre-processing and analysis of the fcMRI data has been published previously^36,42^. To directly compare cortico-cortical functional and genetic correlations, matrices were transformed into a one-dimensional array of unique pairs and then correlated (Pearson’s *r*). To characterize within- versus between-network differences in gene expression, correlations among regions within the same network were averaged (Fig. 2b, on-diagonal) separately for each donor. Between-network correlations (Fig. 2b, off-diagonal) were averaged by network as well, resulting in six between-network estimates for each network and each donor.

### Striatal functional connectivity and gene co-expression

Patterns of cortico-striatal functional connectivity were quantified using fcMRI data from 1000 subjects analyzed by Choi et al.^9,42^. Pearson’s correlations between the time series of each voxel in the striatum and each vertex in the cortex were calculated across the left and right hemisphere (*n* = 10,242 vertices per hemisphere). The average correlation between each functional striatal region to each spatially contiguous cortical parcel was then calculated, resulting in 114 cortical correlations for each striatal network (Supplementary Data 1), using the more fine-grained 17-network cortical parcellation to provide increased spatial resolution. Correlated gene expression between cortex and striatum was estimated using 4912 genes that displayed significant positive differential expression across cortical networks across all six AHBA donors. The default, control, limbic, ventral attention, and somato/motor striatal sub-regions from the population atlas of Choi et al.^9^ were examined; visual and dorsal sub-regions were not examined due to lack of available data. In the cortex, 73 regions that contained samples from at least two donors were examined (Supplementary Fig. 2). Log2 mean-normalized expression within each striatal sub-region and each cortical region were estimated within each subject correlated using Spearman’s *ρ*, Fisher-transformed, and then averaged.

### Cellular gene expression and enrichment analyses

Within AHBA data, 505 overlapping genes were positively differentially expressed within both limbic cortex and striatum. To characterize whether these genes were over-represented among particular cell types, data from Zhang et al.^48^ were used to provide information about the relative expression of genes within astrocytes, neurons, oligodendrocyte precursor cells, newly formed oligodendrocytes, myelinating oligodendrocytes, microglia, and endothelial cells in mouse cortex. The human gene set was converted to mouse homologs using the biomaRt package in R^83^. Three hundred and eighty-eight out of the 505 limbic-associated genes possessed corresponding homologs and cellular expression information. Cellular enrichment for a gene was calculated by comparing the cell type with the highest expression to the one with the second-highest expression value (i.e., fold change). A gene was considered enriched in a cell type if this log2 fold change was > 1.5. We further examined cellular enrichment using data from Doyle et al.^84^, which measured gene expression in 24 distinct cell types in the mouse. Biological replicates in each cell type category were averaged before using Weighted Gene Coexpression Network Analysis^85^ to cluster genes with similar profiles of expression across all of the cell types (Supplementary Fig. 5). All enrichment analyses were conducted using the online ToppGene Suite^38^.

### Laminar expression of limbic network genes

As gene co-expression is associated with structural connectivity^31,86^, we examine whether the 505 limbic network-associated genes were particularly expressed in deeper cortical layers that preferentially possess cortico-striatal projection neurons. Cortical microarray across the OFC and ACC using data from Bernard et al.^28^ were analyzed. Three hundred and thirty of the 505 limbic genes possessed primate homologs identified by Bakken et al.^47^. Expression data from layers 2, 3, 5, and 6 were available. Differential expression between layers 2/3 and layers 5/6 was calculated using *limma* and FDR-corrected *p* ≤ 0.05 significance threshold was applied. Expression values were then hierarchically clustered using a Euclidean distance dissimarilty function and plotted in R.

### Data availability

Analysis code is freely available for download (https://github.com/HolmesLab/CORTICOSTRIATAL_HOLMES). A Supplementary Data file provides complete gene lists and output of differential expression, fcMRI, gene correlation, and enrichment analyses.

## Electronic supplementary material

Supplementary Information

Description of Additional Supplementary Files

Supplementary Data 1
