# Supplementary material for: Gene expression links functional networks across cortex and striatum
Source: Nat Commun. 2018 Apr 12;9:1428. doi: 10.1038/s41467-018-03811-x (PMC5897339; doi:10.1038/s41467-018-03811-x)
Supplement: Supplementary file 1 — Supplementary Information [file 41467_2018_3811_MOESM1_ESM.pdf]

SUPPLEMENTARY INFORMATION: Gene expression links functional networks across cortex and striatum

Kevin M Anderson[1], Fenna M Krienen[2], Eun Young Choi[3], Jenna M Reinen[1], B T Thomas Yeo[4,5], Avram J Holmes[1,5-7*]

[1]Department of Psychology, Yale University, New Haven, Connecticut 06520

[2]Department of Genetics, Harvard Medical School, Boston, Massachusetts 02114

[3]Department of Neurosurgery, Stanford University, Stanford, CA 94305

[4]Department of Electrical & Computer Engineering, Clinical Imaging Research Centre, Singapore Institute for Neurotechnology & Memory Network Programme, National University of Singapore, 117456 Singapore

[5]Athinoula A. Martinos Center for Biomedical Imaging, Massachusetts General Hospital, Boston, Charlestown, Massachusetts 02129

[6]Department of Psychiatry, Yale University, New Haven, Connecticut 06520

[7]Department of Psychiatry, Massachusetts General Hospital, Boston, Massachusetts 02114

*Corresponding author.

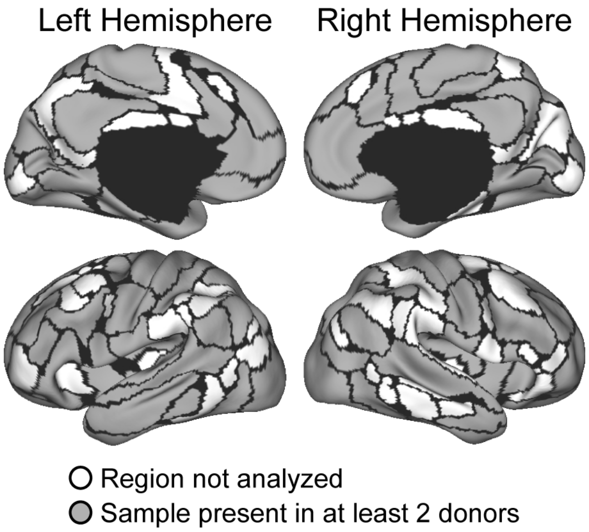

Supplementary Figure 1. Each of the 114 regions from Yeo and colleagues[1] is outlined on the cortical surface. Regions in grey denote the 59 regions containing tissue samples from both donors in the left and right hemisphere. Regions in white denote those that either were not sampled or contain a sample from only one of the two bi-hemispheric donors.

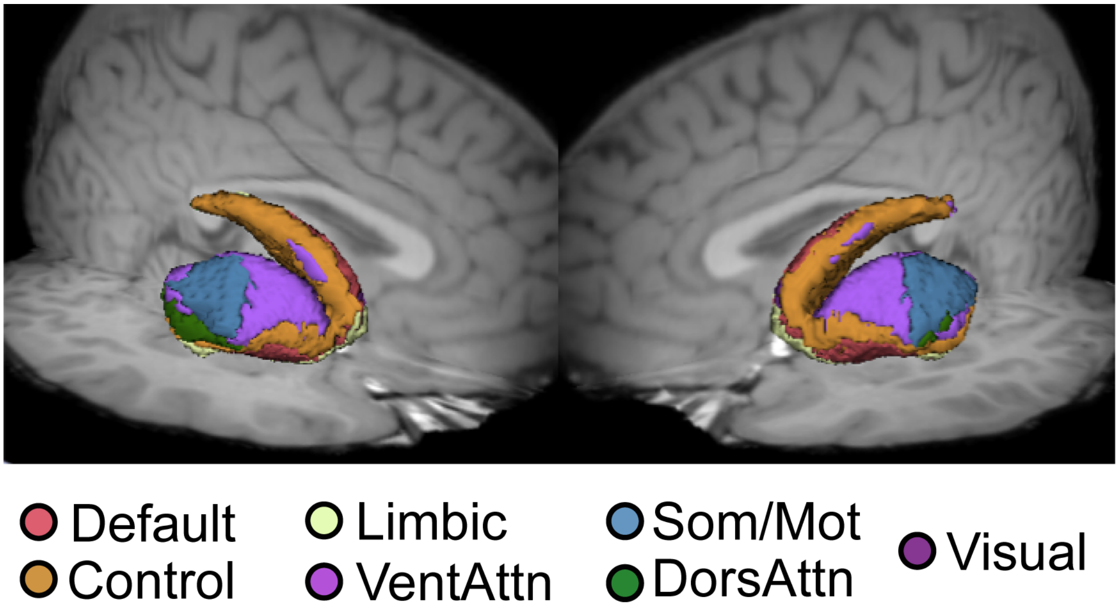

Supplementary Figure 2. 3-D rendering of the 7-network volumetric striatal parcellation of Choi and colleagues[2]. Cream=Limbic, Red=Default, Orange=Frontoparietal Control, Violet=Ventral Attention, Blue=Somatomotor, Green=Dorsal Attention, Dark Purple=Visual.

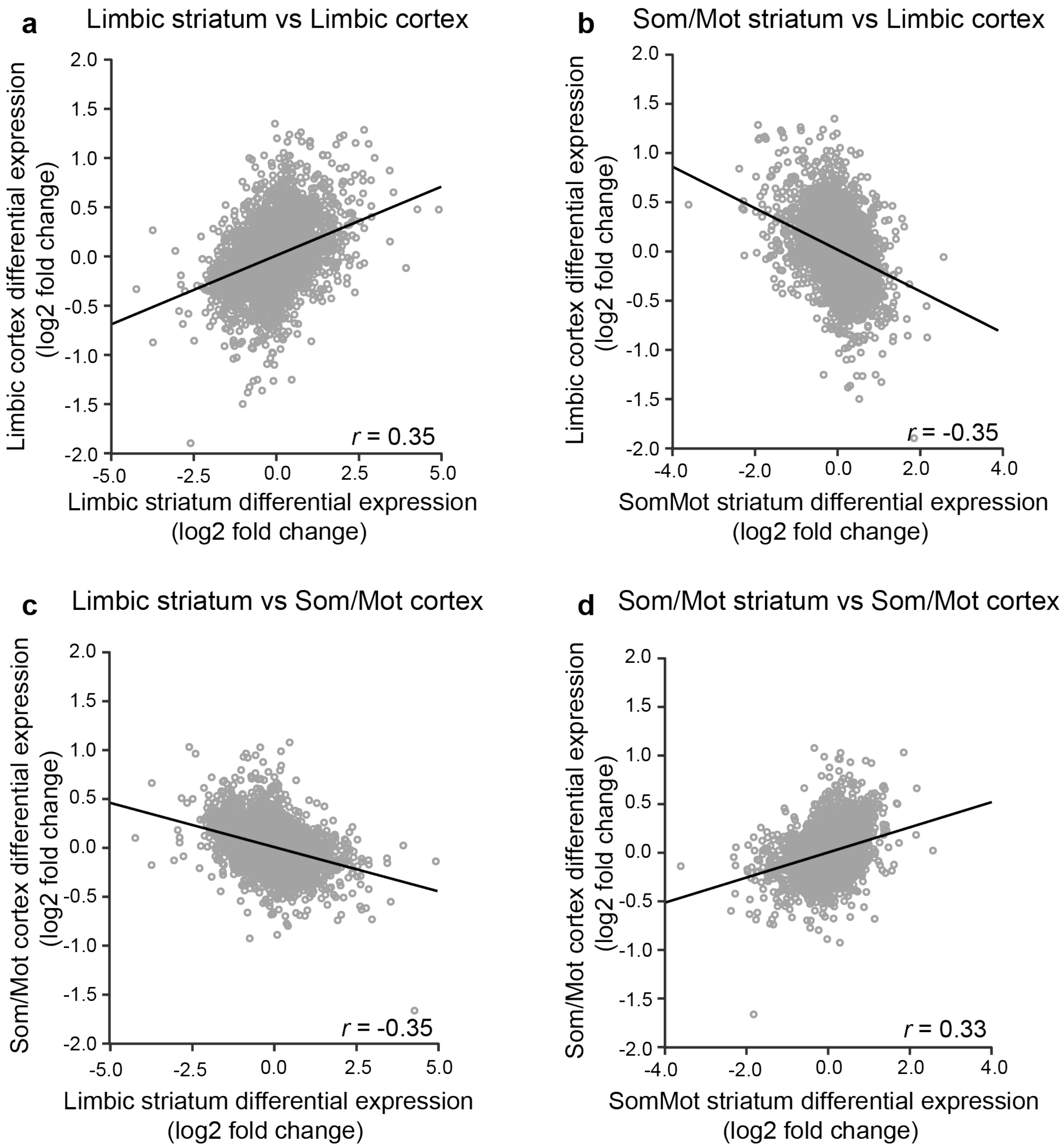

Supplementary Figure 3. Across all genes, relative gene expression in limbic and somato/motor networks were inversely correlated. These data provide evidence that gene co-expression between cortical and striatal regions is not fully attributable to the inclusion of genes that are not expressed in the brain (e.g. 'house-keeping' genes expressed in all cells).

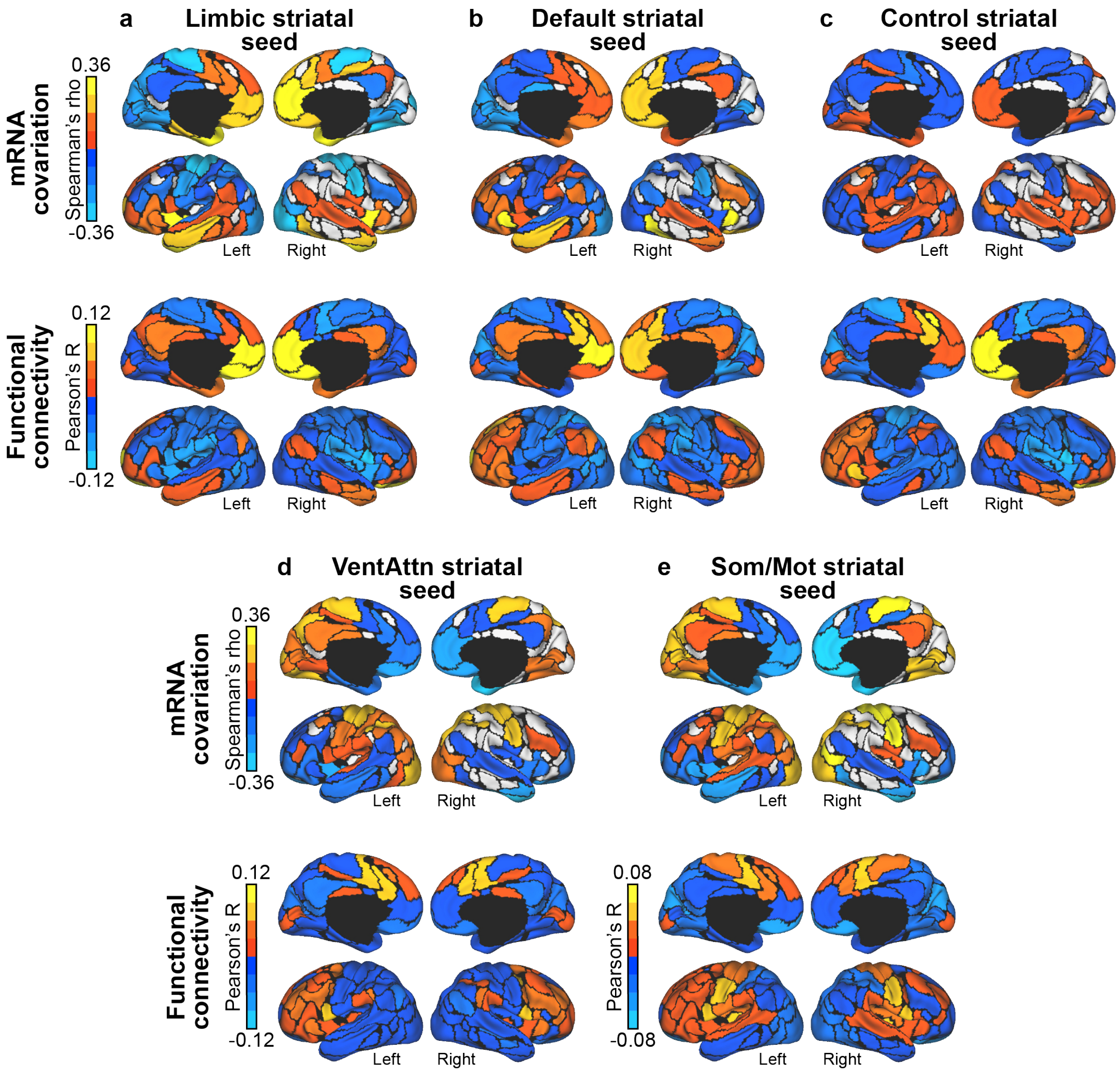

Supplementary Figure 4. Bi-hemispheric unthresholded correlated cortico-striatal gene expression. Genes exhibiting differential expression across cortical networks in all six AHBA donors were examined (n=4,912). The correspondence of fcMRI coupling patterns between the (a) limbic, (b) default, (c) control, (d) ventral attention, and (e) somato/motor regions of striatum with cortex was calculated for the 73 bi-hemispheric cortical parcels using Pearson's r. Gene expression correlations were calculated using Spearman's rho. Regions in gray denote those that do not contain samples from at least two donors.

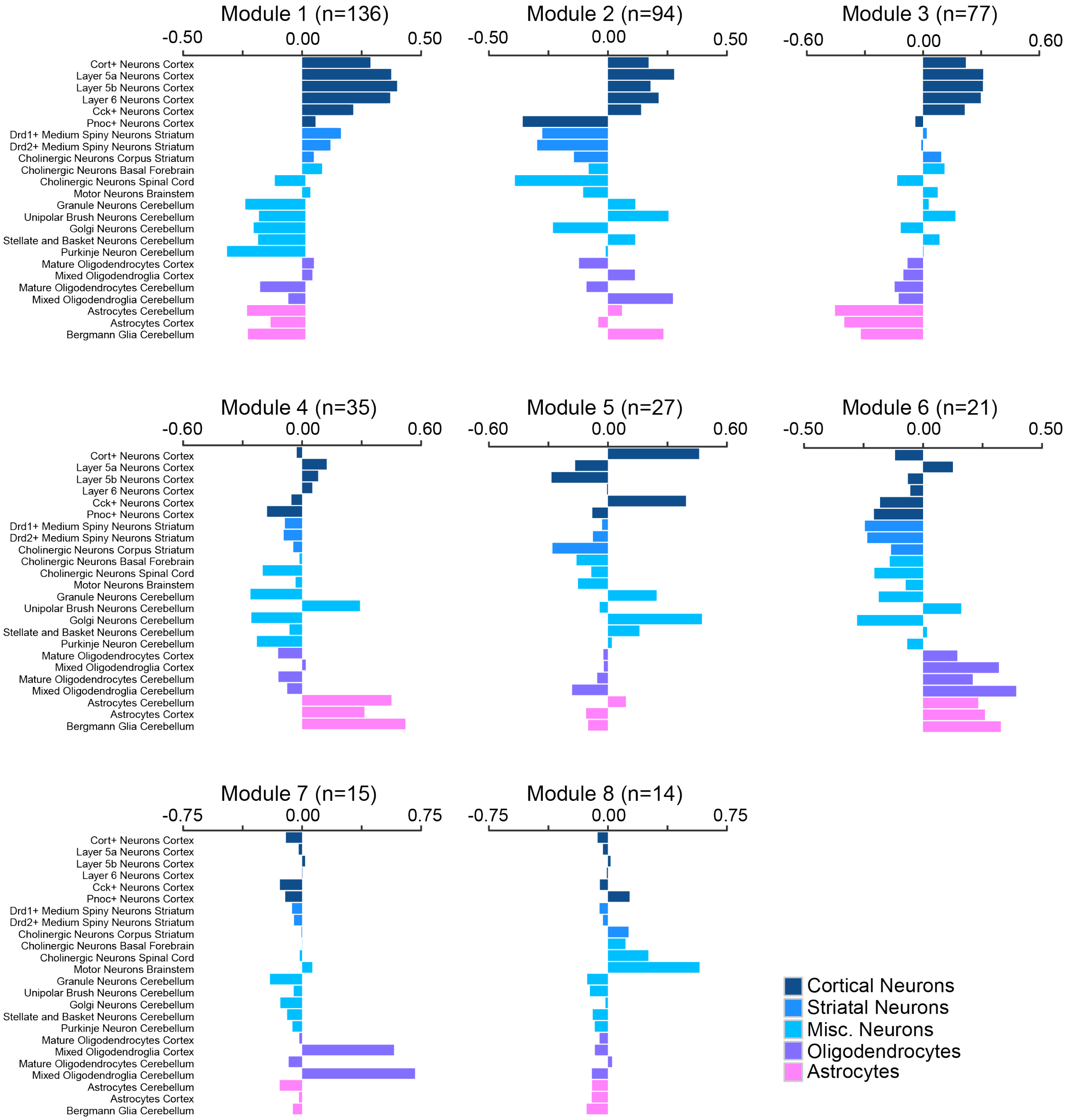

Supplementary Figure 5. The majority of conserved limbic cortico-striatal genes are expressed within neurons. Of the 505 limbic-associated genes, 419 possessed identifiable mouse homologs. Weighted Gene Coexpression Analysis[3] was implemented to identify genes with similar expression profiles across 24 cell classes examined by Doyle and colleagues[4] in *Mus musculus.* Minimum module size was set to groups of 10 genes. Plotted are eigengene expression values for each module. The largest module (Module 1) consisted of 136 genes that were particularly expressed within cortical and striatal neurons.

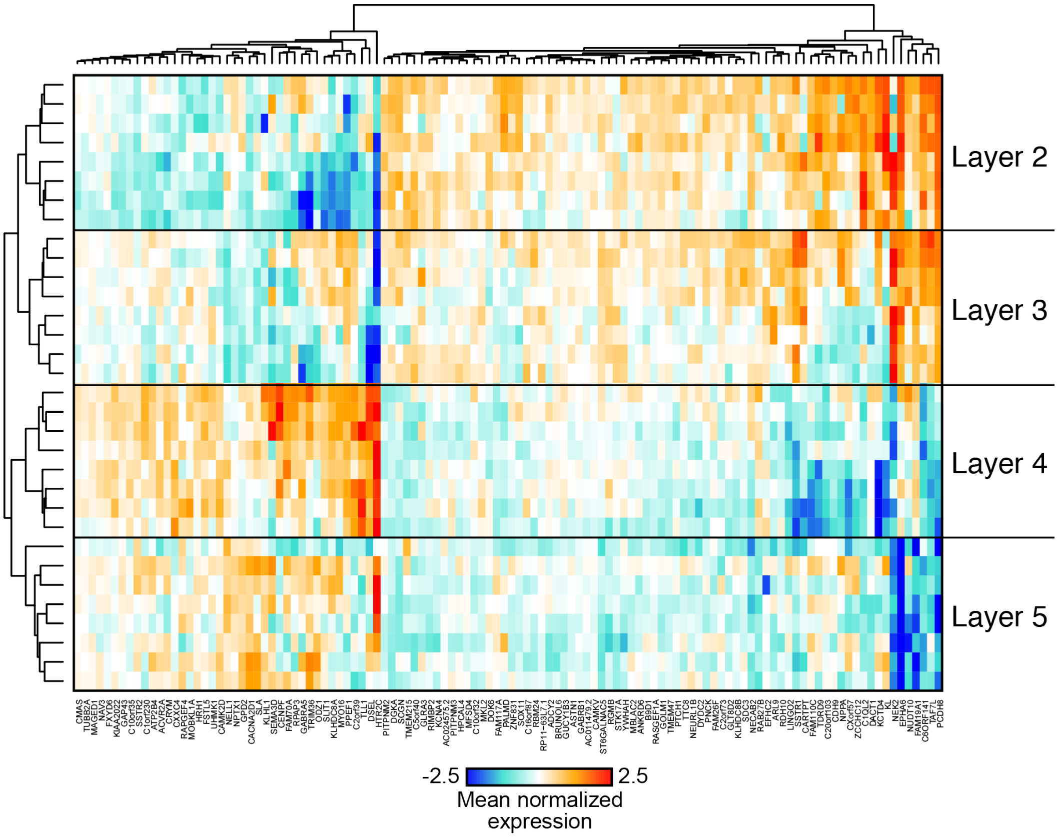

Supplementary Figure 6. Cortico-striatal limbic associated genes are expressed across cortical layers. Of the 505 genes that are consistently expressed in cortico-limbic circuitry, 388 possessed identifiable rhesus macaque homologs. To examine if correlations in limbic cortico-striatal gene expression are driven by cells in certain cortical layers, we used linear modeling to test for expression differences in layer 2/3 versus layers 5/6 of primate anterior cingulate cortex (ACC) and orbitofrontal cortex (OFC) from Bernard and colleagues[5]. Genes were hierarchically clustered according to the similarity their expression across cortical layers. To examine if correlations in limbic cortico-striatal are driven by cortical projection neurons that primarily cluster in deeper layers 5/6, we analyzed laminar-specific gene expression data in the macaque[5]. Of the limbic cortico-striatal gene set with identifiable non-human primate homologs, 30% (count=116) exhibited differential expression between layers 5/6 and 2/3 ($p<0.05$). The bulk of these genes exhibited preferential expression within upper layers 2/3, although 41 genes showed clear increased expression in deeper layers 5/6. The majority of genes (70%) in the cortico-striatal limbic gene set were not biased to a particular laminar depth. Accordingly, convergent cortico-striatal gene expression profiles are likely driven by cells distributed throughout the cortical sheet, rather through isolated lamina or specific classes of cells.

	Age	Ethnicity	Sex
Donor 10021	39	Black/African American	M
Donor 9861	24	Black/African American	M
Donor 12876	57	White/Caucasian	M
Donor 14380	31	White/Caucasian	M
Donor 15496	49	Hispanic	F
Donor 15697	55	White/Caucasian	M

Supplementary Table 1. Demographic information. Donors 10021 and 9861 contain bihemisheric data.

		Donor 9861	Donor 10021	Donor 12876	Donor 14380	Donor 15496	Donor 15697
Visual		38	41	6	35	31	30
Dorsal Attn.		36	19	11	10	15	11
Ventral Attn.		31	24	11	10	14	12
Som/Mot		70	43	16	20	19	17
Control		30	25	11	11	8	11
Default		74	58	30	41	42	33
Limbic		40	33	12	27	14	19
No Overlap		147	118	78	101	71	97

Supplementary Table 2. Counts of cortical samples falling within each functional parcel of the 7-network population atlas of Yeo and colleagues[1].

		Donor 9861	Donor 10021	Donor 12876	Donor 14380	Donor 15496	Donor 15697
ContA		11	15	8	2	6	4
ContB		22	2	6	0	5	5
ContC		2	1	4	6	4	3
DefaultA		21	25	7	9	6	9
DefaultB		26	19	14	22	21	17
DefaultC		1	3	3	2	0	0
DorsAttnA		20	14	1	5	13	4
DorsAttnB		13	5	3	2	6	3
Limbic_OFC		16	19	9	9	7	5
Limbic_Pole		24	15	3	20	9	14
SalVentAttnA		14	10	7	10	7	7
SalVentAttnB		10	11	3	5	6	8
SomMotA		34	24	9	10	11	14
SomMotB		20	12	5	6	6	1
TempPar		13	8	3	2	3	3
VisCent		12	13	0	16	11	9
VisPeri		13	11	2	8	12	11
No Overlap		194	154	88	121	81	113

Supplementary Table 3. Counts of cortical samples falling within each functional parcel of the 17-network population atlas of Yeo and colleagues[1].

		Donor 9861	Donor 10021	Donor 12876	Donor 14380	Donor 15496	Donor 15697
Visual		0	1	0	0	0	2
Dorsal Attn.		0	0	0	0	0	0
Ventral Attn.		9	7	1	4	2	1
Som/Mot		4	2	2	1	1	2
Control		19	15	3	7	4	5
Default		5	1	2	3	2	3
Limbic		2	6	2	3	1	1
No Overlap		9	14	6	6	8	4

Supplementary Table 4. Counts of striatal samples falling within each functional parcel of the 7-network population atlas of Choi and colleagues[2].

		Donor 9861	Donor 10021	Donor 12876	Donor 14380	Donor 15496	Donor 15697
ContA		0	0	0	1	0	1
ContB		4	4	1	1	1	2
ContC		0	0	0	0	0	0
DefaultA		5	1	2	1	0	2
DefaultB		0	0	0	2	0	0
DefaultC		0	0	0	0	0	0
DorsAttnA		0	0	0	0	0	0
DorsAttnB		0	0	0	0	0	0
Limbic_OFC		2	6	2	3	1	1
Limbic_Pole		0	0	0	0	0	0
SalVentAttnA		7	5	0	1	0	0
SalVentAttnB		19	13	3	8	7	4
Som/MotA		1	2	2	1	1	2
Som/MotB		1	0	0	0	0	0
TempPar		0	0	0	0	0	0
VisCent		0	1	0	0	0	1
VisPeri		0	0	0	0	0	1
No Overlap		9	14	6	6	8	4

Supplementary Table 5. Counts of striatal samples falling within each functional parcel of the 17-network population atlas of Choi and colleagues[2].

SUPPLEMENTARY REFERENCES

1. Yeo, B. T. T. *et al.* The organization of the human cerebral cortex estimated by intrinsic functional connectivity. *J. Neurophys.* **106,** 1125–1165 (2011).
2. Choi, E. Y., Yeo, B. T. T. & Buckner, R. L. The organization of the human striatum estimated by intrinsic functional connectivity. *J. Neurophys.* **108,** 2242–2263 (2012).
3. Langfelder, P. & Horvath, S. WGCNA: an R package for weighted correlation network analysis. *BMC Bioinformatics* **9,** 559–13 (2008).
4. Doyle, J. P. *et al.* Application of a translational profiling approach for the comparative analysis of CNS cell types. *Cell* **135,** 749–762 (2008).
5. Bernard, A. *et al.* Transcriptional architecture of the primate neocortex. *Neuron* **73,** 1083–1099 (2012)
