# Supplementary material for: Gene expression links functional networks across cortex and striatum
Source: Nat Commun. 2018 Apr 12;9:1428. doi: 10.1038/s41467-018-03811-x (PMC5897339; doi:10.1038/s41467-018-03811-x)
Supplement: Supplementary file 2 — Description of Additional Supplementary Files [file 41467_2018_3811_MOESM2_ESM.pdf]

Description of Additional Supplementary Files

File Name: Supplementary Data 1
Description: Sheets are described individually below.

1. AHBA_n4_diffExpr Output of the cortical differential expression analyses for each network, in the 4 AHBA donors with left-hemisphere only data. The data in this tab can be used to create the gene list given in "n4_cortical_DEX_genes".

2. AHBA_n4_diffExpr_geneList 2,664 unique genes that were positively differentially expressed across cortical networks of the 4 AHBA donors with left-hemisphere only data. The statistical threshold for differential expression corresponded to FDR B&H $p<0.01$ (i.e. no minimum fold change threshold was applied), although only positively differentially expressed genes were examined.

3. AHBA_n6_diffExpr_Cort Output of the cortical differential expression analyses for each network, in all 6 AHBA donors.

4. AHBA_n6_diffExpr_Cort_geneList Unique genes that were positively differentially expressed across cortical networks of all 6 AHBA donor. The statistical threshold for differential expression corresponded to FDR B&H $p<0.01$ (i.e. no minimum fold change threshold was applied), although only positively differentially expressed genes were examined.

5. AHBA_n6_diffExpr_Cort_Counts Significant differentially expressed genes in the 6 AHBA donors, grouped by cortical network

6. AHBA_n6_diffExpr_Striatal Output of the striatal differential expression analyses for each network, in all 6 AHBA donors.

7. AHBA_n6_diffExpr_Striatal_Count Significant differentially expressed genes in the 6 AHBA donors, grouped by striatal network.

8. AHBA_CortStriat_Overlap Genes that are differentially expressed within both the cortical and striatal aspects of a given cortico-striatal network.

9. ToppGene_limbic_n505 Enrichment output from "https://toppgene.cchmc.org/enrichment.jsp" of the 505 genes that are positively differentially expressed in both limbic cortex and limbic striatum.

10. ToppGene_sommot_n108 Enrichment output from "https://toppgene.cchmc.org/enrichment.jsp" of the 108 genes that are positively differentially expressed in both som/mot cortex and som/mot striatum.

11. mrna_corrmat
The cortico-cortical transcriptional correlations across 59 regions that contained at least 2 samples from each bi-hemispheric AHBA donor (10021/9861). These values were used to produce Figure 1C. The genes examined correspond to the 2,664 genes documented in "AHBA_n4_diffExpr_geneList".

12. fcMRI_corrmat The cortico-cortical resting-state correlations across 59 regions that contained at least 2 samples from each bi-hemispheric HBA donor. Data were from 1000 healthy adult subjects used by Yeo and colleagues (2011).

13. GTEx_striatal_diffExpr Differential expression of GTEx striatal data comparing Nucleus Accumbens samples to putamen and caudate samples.

14. Brainspan_cortLimbic_diffExpr Differential expression of Brainspan cortical data comparing limbic cortex to 8 comparison regions.

15. Brainspan_cortSomMot_diffExpr Differential expression of Brainspan cortical data comparing som/mot cortex to 7 comparison regions.

16. Blueprint_striatal_diffExpr Differential expression of NIH Blueprint atlas striatal data comparing macaque Nucleus Accumbens samples to putamen and caudate samples.

17. Bernard_cortLimbic_diffExpr Differential expression of Bernard macaque cortical data comparing macaque limbic cortex to the rest of the brain.

18. Bernard_cortSomMot_diffExpr Differential expression of Bernard macaque cortical data som/mot cortex to the rest of the brain.

19. Limbic_cellType Genes out of the limbic network genes that show 1.5 log2 enrichment in one of 7 cell types in mouse cortex, using data from Zhang et al. 2014.

20. Limbic_layerSpecific Genes out of the 394 limbic network genes that are enriched in layers 2/3 vs 5/6 in macaque ACC and OFC ($q<0.05$). Negative logFC fold change indicates greater expression in layers 5/6.

21. MRNA_cortstriat_cors Spearman correlation of gene expression for each striatal seed region and each of 73 cortical regions, using the gene set in 'n6_cortical_DEX_genes'

22. rsfcMRI_cortstriat_cors Pearson correlations between each striatal seed region and each of 73 cortical regions, using resting-state data from 1000 healthy adult individuals (Choi et al., 2012).
